# Glycosylation Alterations in Cancer Cells, Prognostic Value of Glycan Biomarkers and Their Potential as Novel Therapeutic Targets in Breast Cancer

**DOI:** 10.3390/biomedicines10123265

**Published:** 2022-12-15

**Authors:** Luka Peric, Sonja Vukadin, Ana Petrovic, Lucija Kuna, Nora Puseljic, Renata Sikora, Karla Rozac, Aleksandar Vcev, Martina Smolic

**Affiliations:** 1 Department of Oncology, University Hospital Osijek, 31000 Osijek, Croatia; 2 Department of Pharmacology and Biochemistry, Faculty of Dental Medicine and Health Osijek, Josip Juraj Strossmayer University of Osijek, 31000 Osijek, Croatia; 3 Department of Pharmacology, Faculty of Medicine, Josip Juraj Strossmayer University of Osijek, 31000 Osijek, Croatia; 4 Pediatric Clinic, University Hospital Osijek, 31000 Osijek, Croatia; 5 Department of Dental Medicine, Faculty of Dental Medicine and Health Osijek, Josip Juraj Strossmayer University of Osijek, 31000 Osijek, Croatia; 6 Health Center Osijek-Baranja County, 31000 Osijek, Croatia; 7 Department of Anatomy, Histology, Embryology, Pathological Anatomy and Pathological Histology, Faculty of Dental Medicine and Health, Josip Juraj Strossmayer University of Osijek, 31000 Osijek, Croatia; 8 Department of Pathophysiology, Physiology and Immunology, Faculty of Dental Medicine and Health Osijek, Josip Juraj Strossmayer University of Osijek, 31000 Osijek, Croatia

**Keywords:** breast cancer, glycomics, biochemical markers

## Abstract

Although we are lately witnessing major improvements in breast cancer treatment and patient outcomes, there is still a significant proportion of patients not receiving efficient therapy. More precisely, patients with triple-negative breast cancer or any type of metastatic disease. Currently available prognostic and therapeutic biomarkers are not always applicable and oftentimes lack precision. The science of glycans is a relatively new scientific approach to better characterize malignant transformation and tumor progression. In this review, we summarize the most important information about glycosylation characteristics in breast cancer cells and how different glycoproteins and enzymes involved in glycosylation could serve as more precise biomarkers, as well as new therapeutic targets.

## 1. Introduction

Breast cancer (BC) is the most common malignant disease in women and one of the top three malignancies across the globe [1]. Over the last few years, BC treatment has advanced significantly, with endocrine treatment, anti-HER2 (human epidermal growth factor receptor 2) antibodies and chemotherapy, which make the therapeutic backbone, depending on the clinical tumor subtype. BC in its early stages is thought to be curable [1], however, in advanced stages patient prognosis is oftentimes poor and better understanding of BC tumor biology and new treatment options are necessary.

The ability to classify patients with BC cancer based on the tumor’s genetic subtype and then steer them toward appropriate, specialized, targeted therapy has become an appealing approach. Although survival rates have improved in recent years, treatment is still complicated and time-consuming, and there are still many fatalities [2,3], especially among patients with triple-negative BC (TNBC) [4]. TNBC is an aggressive subtype of BC characterized by lack of estrogen receptor (ER), progesterone receptor (PR) and absence of HER2 [5]. Immunotherapy has recently emerged as a new treatment option for TNBC, but to advance the clinical development of immunotherapy in TNBC, we need to learn more about mechanisms of response and resistance to it. Although there is no single cure for each type of BC, identifying alterations in glycobiology and glycosylation might tremendously aid us in developing an individualized approach, identifying novel therapeutic targets, and predicting the treatment response or resistance. Glycans and glycoproteins have been studied in the progression of BC and other malignancies for decades [6]. Changes in glycosylation provide new insight into the characteristics of BC. In addition to glycoproteins, we will also focus on other glycosylated compounds (glycoconjugates) such as glycosphingolipids (GSLs) and proteoglycans. As a component of the cell membrane, GSLs could also be a potential biomarker and a therapeutic option. The functions of GSLs are very diverse and are extensively discussed in the literature [7,8]. Certain alterations in glycosylation are linked to a patient prognosis in BC, suggesting that they might be used as a predictive biomarker in the future [9]. Glycosylation alterations also lead us to novel drug action sites, enhancing responsiveness to therapy, and evaluating the impact of a certain therapy, such as chemotherapy or radiation [10,11,12]. In this review, we will go over the basics of protein glycosylation, which is the most significant post-translational protein process, as well as significant glycans and other glycoconjugates showing the most recent results on how alterations in these processes in BC are significant in diagnostics, progression evaluation and response to different therapeutic modalities. Alterations in glycosylation, a hallmark of tumor cells, lead to the formation of aberrant carbohydrate molecules known as tumor-associated carbohydrates (TACAs) [13,14,15]. TACAs play a significant part in scientific research, which can greatly influence clinical practice. These carbohydrate molecules are present in substantial concentrations in a variety of cancer types, making them fascinating targets for treatment and drug development, as well as the possibility of utilizing these structures as biomarkers for both diagnostic and therapeutic response evaluation. One of the better-known biomarkers that are widely accepted as a diagnostic method in clinical practice belong to the TACAs group of molecules.

Some TACAs are already valuable biomarkers in clinical practice, such as carbohydrate antigen 19-9 (CA19-9) (a biomarker in pancreatic cancer) and serum tumor marker carbohydrate antigen 72-4 (CA72-4) in gastric cancer but there are also numerous TACAs related to breast cancer [16,17].

**Figure 1:**
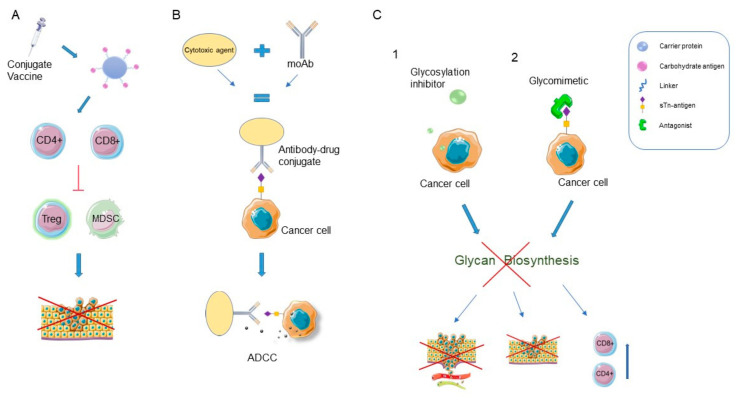
Glycan-based therapies. (**A**) Carbohydrate vaccine induces a cytotoxic T lymphocyte response and anti-tumor immunity by reducing the function of immunosuppressive cells (Treg and MDSC) and inhibits the proliferation of cancer cells. (**B**) Antibody drug conjugates (ADCs) are targeted agents that combine less specific chemotherapeutic agents with glycan-specific antibodies. Monoclonal antibodies (moAbs) promote antibody-dependent cellular cytotoxicity (ADCC). (**C1**) Glycosylation inhibitors are small molecules that can interfere with metabolism of precursors or intracellular activities; (**C2**) Glycomimetics selectively block glycan receptors on the surface of cancer cells. Glycosylation inhibitors and glycomimetics interfere with glycan biosynthesis, preventing cancer cell adhesion to endothelial cells, metastasis, and enhancement of cytotoxic T cell immunity. CD4+—Helper T cell; CD8+—cytotoxic T lymphocyte; Treg—regulatory T cell; MDSC—myeloid-derived suppressor cell. Figure created with Servier Medical Art, https://smart.servier.com/ accessed on 20 July 2022.

Finally, we will review latest promising treatment targets associated with glycosylation (Figure 1).

## 2. Glycosylation

Despite major advances and accumulating knowledge regarding cellular mechanisms and novel anti-cancer strategies, neoplastic diseases continue to be one of the main foci of biomedical research, with accurate and timely diagnosis along with precise treatment as one of the most important challenges in modern medicine [18,19]. Neoplastic transformation involves major cellular alterations impacting proliferation, survival and behavior of affected tissue. Multi-omics approaches presents a novel framework that integrates multiple omics data, such as genomics, epigenomics, transcriptomics, proteomics, metabolomics, pharmacogenomics, glycomics and glycoproteomics, providing integrative approaches for understanding the molecular and clinical features of cancer and eventually the development of successful precision strategies in cancer therapy [20,21]. Glycomics defines the complete set of glycans, referred to as glycome, produced by a cell or tissue, while glycoproteomics describes glycome on the cellular proteome, meaning it determines glycosylated sites on each glycoprotein and identifies and quantifies glycan structures [22].

In this section, we will be focusing on glycans due to their gaining momentum in cancer research. Recently, glycosylation has emerged as a key process of neoplastic transformation, directly impacting cell proliferation, survival, immune modulation, and metastasis [23].

Glycosylation is one of the most diverse and complex post-translational modification of proteins. Briefly, this process involves enzymatic modifications of proteins, creating glycoproteins, hence, altering their biological functions and amplifying the proteome. Glycosylation of proteins is defined as an attachment of glycans (polysaccharide chains) to the proteins through an enzymatic reaction mainly in endoplasmic reticulum and Golgi apparatus. About 200 glycosyltransferase enzymes are responsible for glycosylation processes. The two most common types of protein glycosylation are known as *N*-glycosylation and *O*-glycosylation, depending on whether glycans are linked to nitrogen or oxygen atoms of amino acid residues of the protein. These processes are achieved through specific ways—*N*-linked to asparagine (Asn), *O*-linked to the hydroxyl groups of serine (Ser), threonine (Thr) or tyrosine (Tyr).

Human glycome is built from 10 monosaccharides derived from activated donor sugar nucleotides or dolichol (Dol)-linked donors. The most common monosaccharides found in glycans are *N*-acetylgalactosamine (GalNAc), fucose (Fuc), mannose (Man), glucose (Glc), galactose (Gal), *N*-acetylglucosamine (GlcNAc) and sialic acids, in humans mostly *N*-Acetylneuraminic acid (Neu5Ac) [24]. Ensemble of glycoproteins, as well as other glycoconjugates at the cell surface that we will focus on in this paper, is called the glycocalyx. The clinical significance of this process is that these unique alterations in neoplasm glycosylation serve as a distinct feature of cancer cells and therefore provide novel diagnostic and therapeutic targets [23].

Due to the complexity of malignant transformation and importance of glycans in almost all pathophysiological processes, oncogenesis is irrefutably accompanied by altered glycosylation. Alterations in glycosylation have been recognized as a hallmark of oncogenesis, with a significant amount of in vitro and in vivo studies showing correlations between glycosylation alterations and cancer prognosis, supporting the role of glycan changes as critical to tumor cell behavior and increasing the interest in glycans and glycosylation alterations as biomarkers and potential targeted therapy. Changes in glycosylation in cancer cells are various, with the most common including decreased or enhanced expression of certain glycans and increased expression of incomplete or truncated glycans. Epigenetic changes in glycosyltransferases in cancer cells also result in completely novel glycan structures that appear to be cancer cells’ mechanism of avoiding host immune response. Studies on enzymes associated with aberrant glycosylation as cancer biomarkers have shown that in tumor cells, the activities of sialyl- and fucosyltransferases are increased [25,26], while the activities of galactosyl- and *N*-acetylglucosaminyltransferases are reduced compared to normal cells fucosylation was shown to be more pronounced in BC cells [25,27,28]. Glycosylation alterations will be briefly explained through this article with focus on their potential in clinical practice. Certain glycans are already valuable biomarkers in clinical practice; however, the significance and the need for further research will aid better understanding in tumorigenesis. Therefore, improved diagnosis, prognosis and therapeutic options is irrefutable [29,30].

## 3. Glycans as Biomarkers of Breast Cancer Aggressiveness

The role of glycosylation in BC has recently become an interesting and important research topic because it is believed that it can greatly help clinicians in choosing the right treatment and predict response to therapy and patient prognosis. However, the role of glycosylation, or more precisely the change in composition of glycocalyx, was actually linked to BC about 70-years ago [31]. In addition, throughout history, it was found that the identification and analysis of glycosylation changes can be used for the discovery of relevant BC biomarkers because a variety of glycans and glycoconjugates are exhibited both in cancer tissue and serum. In general, the role of biomarkers in oncology is to improve early disease detection (screening method), for risk assessment and to predict response to treatment and monitor disease progression [32]. The potential of different glycans and other glycoconjugates, involved in glycosylation to become cancer biomarkers was discovered once it was noticed that they were expressed differently in malignant as compared to normal cells. Because of the potential for the development of novel diagnostic and therapeutic approaches, separating normal cells from malignant cells plays a significant role in scientific research. One of the ways to distinguish a healthy (normal) cell from a tumor (malignant) cell is by searching for abnormal varieties, forms, and quantities of carbohydrate structures on cancer cell surfaces.

A significant number of studies show the importance of TACAs in breast cancer such as Thomsen-nouvelle antigen (Tn), sialyl-Thomsen-nouvelle antigen (sTn), Thomsen–Friedenreich antigen (TF) and sialyl-LewisX (sLeX) [16,33,34,35,36,37,38]. Tn-antigen is expressed in almost all breast cancer tissues, in contrast to normal tissue, where it can rarely be observed. Studies have also suggested that Tn-antigen is linked to more aggressive tumor behavior because it is linked to lower overall survival (OS) and higher recurrence risks [38,39]. STn antigen is also not often found in healthy tissue, but studies have shown that most carcinomas exhibit it at varying frequencies. Current knowledge obtained regarding STn antigen shows that up to a maximum of 30% of breast cancers are sTn-positive. It is also associated with poorer outcomes and increased aggression, notably poorer patient survival rates [37,40]. TF antigen, as well as the previously mentioned two antigens are typically highly expressed in breast cancer. More research is needed to determine the association between aggressiveness, patient prognosis, and TF antigen, due to inconsistent reports regarding the relationship to overall patient survival or metastatic potential [37,41]. High expression of sialyl-Lewis antigens has been found in a number of malignancies and sLeX overexpression has been associated with poor survival [9,42,43,44]. This is supported by the finding that high serum levels of sLeX were found in invasive ductal BC and invasive micropapillary carcinoma [44], but not in ductal carcinoma in situ and that these patients had shorter progression-free period and OS [9]. SLeX antigen in BC patients has also been linked to the development of cutaneous metastases, which are generally rare and associated with more aggressive BC subtypes [45]. The most likely explanation for their role in malignant spread is because these antigens are ligands for adhesion receptors expressed on activated endothelial cells, thereby promoting penetration into distant tissues [25,45]. These findings make sLeX a promising biomarker candidate for identification of more aggressive BC types which would require appropriate treatment. A study examining levels of specific serum *N*-glycans by isolating circulating tumor cells (CTCs) found that glycans containing sLeX epitopes (A2F1G1, A3F1G1, A4F1G1, and A4F2G2) were significantly increased in patients with metastatic breast cancer compared to healthy patients [46]. Other studies on the glycoprotein Mucin 1 (MUC1), known to be aberrantly glycosylated and highly expressed in various cancers, found that aberrant MUC1 expresses oligosaccharides such as sLex. High levels of MUC1-carrying sLex correlate with a poor prognosis in patients with some types of carcinoma [47]. Another tumor-associated antigen Lewis C antigen (LeC) has been shown to play a significant role in BC. A number of studies show that the disaccharide LeC is a potential biomarker, as patients with BC have lower levels of nAbs (natural antibodies) of immunoglobulin M against LeC compared to healthy patients. These studies suggest that anti-LeC nAbs are involved in cancer surveillance and are believed to be cytotoxic to cancer tissues [48].

Furthermore, when identifying potential biomarkers of breast cancer, we must mention *N*-glycans. *N*-glycans are essential in cancer development and growth. Interestingly, as early as in stage 0 of BC, *N*-glycosylation of IgG is notably aberrant and as such detectable in the serum [49]. *N*-glycomic signals in general are more common in the early stages of cancer than later. Changes in serum protein glycosylation have been observed in a variety of malignancies, particularly breast cancer, indicating that serum glycans might be useful biomarkers for BC [50,51,52]. According to research by Sae Byul Lee et al., MALDI-TOF *N*-glycomic analysis of BC has greater diagnosis effectiveness than traditional procedures such as mammography or ultrasound [52]. This is supported by recent findings showing the abundance of high-mannose glycans Hex6HexNAc2, Hex7HexNAc2, Hex9HexNAc2, and Hex10HexNAc2 which were much higher in BC patients than in healthy people [52,53,54]. This research and associated findings reaffirm the relevance of *N*-glycans as possible biomarkers for breast cancer diagnosis. These antigens are already being investigated as a potential glycan-based targeted therapy [55] or immunotherapy [56].

Another glycoconjugate, glycosphingolipids, show involvement in cell interaction, differentiation, control, adhesion, and cell proliferation as one of their most significant functions. In the literature, the particular forms of GSLs, often referred to as tumor-associated antigens are more often described and present in cancer tissue than in healthy tissue [7,8]. Signaling pathway of globo-series GSLs is one of the most important pathways associated with breast cancer and GSL [57]. Globo-H (globohexaosylceramide), SSEA3 (stage-specific embryonic antigen 3), and SSEA4 (stage-specific embryonic antigen 4) are the three most significant GSLs from the aforementioned series. The regulation of cell proliferation results from a specific interaction between GSL and signaling pathways, which might make tumors more aggressive and hence more likely to develop metastases. GSLs contribute significantly to tumor development and are a prospective target for cancer therapy. In particular, the identification of tumor-associated GSL antigens that have been utilized in the creation of anticancer vaccinations has recently attracted attention. Results revealed that fluorine-modified *N*-acyl Globo H conjugates can increase IgG antibody titers which can identify the globo H antigen on cancer cells and thereby take part in cancer cell eradication. This suggests possibilities for immunogenicity and development of new vaccines that could aid in the suppression of cancer cells [58].

The literature suggests another potential biomarker of a more aggressive BC subtype, 2,3-sialic acid, because it was found to be overexpressed in higher grades of BC and metastatic disease [59]. This finding was complemented by the upregulation of mediators of malignant invasion: matrix metalloproteinase-2 and -9, cyclooxygenase-2 and β1-integrin by the α2,3-sialyltransferase, ST3Gal III [60].

A significant number of studies have been focusing on changes in the expression of enzymes involved in glycosylation and their potential role as biomarkers. Regarding fucosyltransferase 8, it was discovered that its overexpression potentiates TGF-β (Transforming growth factor-β) and EGF (Epidermal growth factor) signaling and is associated with the aggressive type of BC promoting its spread [61,62]. With regard to α2, 6-sialylation, it was discovered that such integrin glycosylation potentiates BC cell metastatic ability because it impacts cancer cell adhesion to extracellular matrix (ECM) components as well as cell–cell adhesion [25,63]. Sialylation of other structures involved in cell–cell interactions, such as sialyl-Lewis antigens (sialyl-LewisA and sLeX, TF-antigens, sialyl α2,6-lactosaminyl structures and polysialic acids, are all changed in cancer cells [64]. Studies have shown a significant role of *N*-acetylgalactosaminyltransferase 6 (GALNT6) in cancer cells. GALNT6 is an enzyme that controls the first stage of mucin-type *O*-glycosylation and was found to be associated with BC [12]. The underlying role of GALNT6 in tumor progression, on the other hand, has not been completely investigated. In a study conducted via online database analyses and tissue microarrays, it was found that the OS of BC patients with GALNT6 overexpression was poorer than those with low GALNT6 expression [12]. Furthermore, GALNT6 overexpression correlated with higher BC pTNM stages. The mRNA level of GALNT6 has been found to be positively correlated with malignant infiltration of bone marrow in BC patients [65]. This enzyme is later further explained as a potential targeted therapy.

Overall, further research in this area might substantially benefit clinical practice for disease stratification and for choosing the treatment approach in a population of BC patients. An overview of potential biomarkers and treatment targets is given in Table 1.

## 4. The Impact of Glycosylation Changes on BC Treatment Outcome and Its Potential as Aid in the Discovery of New Targeted Therapies

In the final part of our paper, we will look into some molecular mechanisms of glycosylation alterations in cancer cells affecting BC treatment outcome and compounds that are currently being tested as either potential therapeutic target or an evaluating tool for efficiency of currently available oncological therapy in clinical practice, such as radiotherapy. We divided this section into three parts, two of them based on the type of glycosylation in which compounds of interest are involved, with the last part focusing on the current status of glycan-based therapy, its success and perspectives, as well as the possibility of evaluating efficiency of therapy commonly used in oncology, specifically radiotherapy, with glycan biomarkers.

### 4.1. N-Glycosylation 

Due to accumulating knowledge regarding the importance of glycosylation alterations in oncogenesis, modulating glycosylation processes in cancer is a logical potential therapeutic option. As explained prior, *N*-glycosylation is one of the most common protein posttranslational modifications, highly significant in BC. This significance stems largely from HER2, an *N*-glycosylated glycoprotein, overexpressed in many types of cancer, however, especially significant in BC and is already a therapeutic target of the monoclonal antibody trastuzumab as well as other options, such as inhibitors targeting its tyrosine kinase activity [23]. Other potential targets we will discuss in this section mainly include various glycosyltransferases involved in aberrant *N*-glycosylation in cancer cells.

*N*-glycosylation of programmed death-ligand 1 (PD-L1) is necessary for its interaction with PD-1, which subsequently enables immune evasion in tumor microenvironment. In this study on a mouse model, targeting PD-L1 glycosylation sites with an antibody–drug conjugate induced PD-L1 removal from the cell membrane and prevented its further activity, leading to reduced tumor growth in immunocompetent mice [68]. Earlier studies found that β-1,3-*N*-acetylglucosaminyl transferase (B3GNT3) is a key glycosyltransferase that mediates *N*-glycosylation of PD-L1 glycoprotein [69,70]. Immunotherapy as a relatively new mode of therapy in oncology is finding its place in the treatment of highly aggressive TNBC. TNBC contains the greatest frequency of PD-L1-positive cells and the highest mutational index among BC types; however, clinical studies show that immunotherapy response in such patients is still quite limited [39,71]. Therefore, new insights into the mechanisms involved in checkpoint blockade could be the key to more successful treatment of this patient group. The importance of glycosylation in enhancing the function of an immune inhibitory receptor in TNBC is described in the study by Li et al. [68,72]. Given the rate of PD-L1 presence in TNBC cells, targeting this mechanism is thought to bear good potential in TNBC treatment [39,71,72]. Another animal study supported this finding, namely that interference with glycosylation of PD-L1 leads to its loss of function and in turn increases the efficacy of immunotherapy and radiotherapy in TNBC [73]. A combinatorial treatment with epidermal growth factor receptor (EGFR) inhibitor gefitinib and a saccharide analogue, 2-deoxy-D-glucose can enhance antitumor immunity in murine model of TNBC by deglycosylation of PD-L1 [74]. Gene mutations affecting glycosyltransferases lead to significant changes in glycosylation, which are known to be a hallmark of malignant cells. As mentioned in the section regarding biomarkers, this knowledge is now being used in an attempt to develop precise tools for early detection of malignancies, their classification and targeted treatment [75]. One of the well-known changes regarding glycosyltransferases is increased sialylation of transmembrane proteins, a characteristic of different types of malignant cells (including BC) and was found to contribute to cancer metastasis and poor patient outcome [76]. Therefore, multiple in vitro and animal studies with sialyltransferase (ST) inhibitors have been conducted with the attempt to reduce or delay malignant spread [77,78]. An animal study on two ST inhibitors, FCW34 and FCW66 showed that they can suppress the metastatic spread by inhibitory effects on tumor cell proliferation, migration and angiogenesis via modulation of the talin/integrin/FAK/paxillin and integrin/NFκB signaling pathways which resulted from reduced *N*-glycan sialylation [78]. Glycosylation inhibitors and mimetics are relevant potential drugs due to their ability to interfere with biosynthesis of glycans and glycan receptors (Figure 1C) [79].

One of the processes associated with the progression of cancer is an epithelial–mesenchymal transition (EMT), a process in which cells lose epithelial cell properties and acquire those commonly expressed by mesenchymal cells. Thus, during EMT, epithelial cells lose their polarity and intercellular connections and in addition, develop migratory and invasive properties similar to those of mesenchymal cells [80,81]. Therefore, in order to metastasize, cancer cell needs to transform into mesenchymal-like form.

One study showed that glypican-3 (GPC3), a proteoglycan which regulates cell proliferation and survival also contributes to EMT inhibition in BC cells. Interestingly, the same study showed that GPC3 overexpression actually led to a reverse mechanism called mesenchymal–epithelial transition (MET) and was protective in that way [66]. The molecular mechanism and the role of GPC3 in cancer signaling and tumor growth require further research and elucidation [82]. A different study investigated the effect of the flavonoid luteolin on highly aggressive TNBC cells and found that it also inhibited EMT by interacting with the β-catenin pathway [83].

Regarding glycosyltransferases, an in vitro study on human MDA-MB -231 BC cells which were modified to overexpress β4-*N*-acetyl-galactosaminyltransferase 4, discovered that changes in their phenotype occurred in a way that they lost mesenchymal-like characteristics and transformed into epithelial-like by changing their shape into a cobble-stone-like morphology and increasing the expression of E-cadherin. In addition, the cells lost their migratory activity and increased adhesion to extracellular matrix proteins. These changes resulted from expression of LacdiNAc on cell surface glycoproteins and suggest lower migratory potential [81]. Another in vitro study found a connection between changes in glycosyltransferases and EMT.

An enzyme which mediates terminal α2,6-sialylation to *N*-glycans, β-galactoside α2,6-sialyltransferase 1 (ST6GAL1), also plays a role in TGF-β-induced EMT. Its overexpression has been linked with progression in a variety of tumors. On the contrary, silencing *St6gal1* disables EMT and in fact leads to expression of epithelial-like characteristics in the cell culture model [84]. Thus, in cancer cells, the expression of a specific glycosyltransferase and its glycan products is intimately linked to EMT or MET [33,81,84,85]. On the other hand, TGF signaling is widely recognized as a major EMT inducer through activation of the Smad complex that translocates to the nucleus to regulate expression of genes important for BC development and its heterogeneity [86]. Given the above findings about the EMT process, there seems to be a number of potential therapeutic targets that could be explored. EMT is one of the crucial steps in the development of BC metastasis and is worth further attention.

As mentioned before, *N*-glycosylation is a very important post-translational modification and certain changes in this process are characteristic of malignant transformation and further progression of malignant disease. A recently published study by Ščupáková K. et al. performed on tissue samples from deceased patients with metastatic BC found that several *N*-glycans increased sequentially along the metastatic path for each individual, starting with low concentrations in normal breast epithelial tissue, followed by slightly increased amounts in the primary tumor (PT) and lymph node metastases, and strongly elevated in distant metastases. In addition, with the exception of bone metastasis, which had greater levels of fucosylated *N*-glycans, research findings showed that high-mannose *N*-glycans were the most abundant in distant metastases compared to PTs and normal breast tissue. Given the buildup of high-mannose *N*-glycans in PT and metastatic BC in this research, therapies that improve the activity of the mannosidase I group enzymes may be worth studying [11]. Other studies also proved persistently enhanced *N*-glycosylation in primary breast tumors and distant metastases [53,87].

Another interesting avenue worth exploring appears to be the use of statins in BC patients. It is known that *N*-glycans, Golgi remodeling and metabolism are all linked to EMT and oncogenic dissemination in patients with BC. It is also known that metastatic recurrence is the most common cause of death among BC patients [88,89]. Interestingly, according to many retrospective studies, patients who used statins [88,89,90,91] had a decreased risk of postsurgical BC recurrence. A study from 2021 showed that inhibition of mevalonate pathway with fluvastatin lowered the *N*-glycosylation and *N*-glycan-branching, both of which are critical elements of the EMT mechanism and metastatic spread [89]. These results demonstrate that metastatic BC cells rely on the fluvastatin-sensitive mevalonate pathway for dolichol-dependent protein *N*-glycosylation and can be therapeutically targeted by blocking dolichol biosynthesis with inhibition of the conversion of 3-hydroxy-3-methylglutaryl coenzyme A (HMG-CoA) to mevalonate (MVA). This suggests that the use of fluvastatin as an adjuvant medication in BC treatment is worth further investigation [89]. Statins would be worthy choices for repurposing as metastatic prevention medicines since they are currently clinically licensed, affordable, and have acceptable safety profiles that allow long-term usage [91]. The discovery of the mechanism of statin antineoplastic function also opens the door to the development of new actionable biomarkers that may be used to identify individuals who would benefit from such therapy.

Given all of the literature data on association between *N*-glycosylation and BC, it is not surprising that a number of clinical trials have been conducted on agents that in some way target *N*-glycosylation [92]. However, in clinical practice, using this type of treatment is still confronted with major difficulties. Above all, we do not have a complete picture of the glycome in health and illness yet. The development of glycan-targeted treatments will be accelerated by improvements in our capacity to detect altered glycosylation, as well as the number of effective glycan biomarkers. Tumor-associated carbohydrate antigens (TACA) are a main target for cancer vaccination development that function as tumor rejection targets. Vaccine strategy, as shown in Figure 1, enhances the immune response and induces tumor regression and rejection by incorporating antigens essential for cellular transformation, including proven tumor-rejection targets. The proposed mechanism of immune-mediated tumor rejection is primarily associated with the CD8+ T-cell and CD4+ helper T-cell response. Patients with BC have increased numbers of both regulatory T cells (Tregs) and myeloid-derived suppressor cells (MDSC). In response to E75 peptide vaccine, derived from tumor-associated glycoprotein HER2 studies have shown increased CD8+ T-cell and CD4+ helper T-cell, but also decreased regulatory T cells (Tregs) and myeloid-derived suppressor cells (MDSC) as shown in Figure 1A [93]. A phase II/III trial of Globo-H vaccination in BC patients (NCT01516307) did not increase progression-free survival

(PFS) and overall survival; however, it significantly improved PFS and OS in the 50% of patients who developed an immune response to the vaccine with anti-Globo H IgG titers ≥ 1:160 compared with patients with IgG titers ≤ 1:160 and patients in the placebo group [94]. The same vaccination is presently being tested in a phase III study in TNBC (NCT03562637) [95]. Vaccines for cancer therapy improve the immune system’s ability to detect and eliminate antigens. It is worth noting that BC vaccines are both innovative and complex, but we will have to wait to see how safe and effective they will be. Targeted immunotherapy is also progressing with the development of monoclonal antibodies that modulate immune checkpoints for BC (Figure 1B) [93]. Galili et al., found that the anti-Gal antibody recognizes glycosphingolipids with non-reducing terminal Galα1-3Galβ1-4GlcNAc-R structures, whereas it does not bind to a range of closely related glycosphingolipids and could be exploited in clinical practice as cancer immunotherapy [96].

### 4.2. O-Glycosylation

*O*-glycosylation are also associated with malignant disease. As previously mentioned, one of the enzymes associated with glycosylation and the progression of BC is GALNT6 that controls the first stage of mucin-type *O*-glycosylation and was found to be associated with BC [12]. However, the role of GALNT6 in tumor progression is not clear. A study on cell cultures showed that GALNT6 and other GALNTs induced tumor progression through modulation of PI3K/Akt (phosphatidylinositol 3-kinase/protein kinase B) and MAPK/ERK (extracellular signal-regulated kinases/microtubule associated protein kinases) signaling pathways. Specifically, GALNT6-OE boosted Akt phosphorylation in MDA-MB-468 cells, while GALNT6-KD reduced Akt phosphorylation in MDA-MB-231 cells. GALNT6 was also shown to enhance metastatic properties in BC cells by enhancing mucin-type *O*-glycosylation of α2 microglobulin [97]. Altogether, these findings point researchers to a new approach in their investigation of a role of GALNT6 in the development of an oncogenic mechanism which could explain the poor prognosis in some breast neoplasms [12]. Dysfunctional *O*-glycosylation has also been found to be involved in BC progression. Protein from the Ragulator-Rag complex LAMTOR5, also known as hepatitis B X-interacting protein, is required for amino-acid-induced mTORC1 (mammalian target of rapamycin complex 1) activation [98]. LAMTOR5 has been found to be overrepresented in BC where it promotes BC development by co-activation of numerous transcription factors, including Sp1, c-Myc, and LXR [98,99,100]. The excess of truncated *O*-glycans, such as Tnantigen, which is formed by the aberrant start of GalNAc-type *O*-glycosylation, is a typical hallmark of malignancy. In addition, another recent study discovered that LAMTOR5, synergistically with s-Src and c-Jun, increased aberrant *O*-glycosylation and Tn-antigen accumulation through influencing GALNT1 expression and dislocation, which led to BC metastasis. Altogether, the findings of these studies add to understanding of the regulatory mechanism that leads to aberrant *O*-glycosylation initiation, and they suggest that the LAMTOR5/c-Jun/c-Src network might be used as a prognostic biomarker and therapeutic target for metastatic BC [99].

### 4.3. Glycan-Based Therapy—Perspective and Limitations

The introduction of glycans as therapeutics is considered as an important step in developing novel strategies to fight BC. It is important to mention the most recent advances in glycan-based therapy research and their current status in clinical trials. Currently, glycan-based therapy of clinical relevance includes vaccines, glycoconjugate drugs, and glycosylation inhibitors.

Corti et al., provide a valuable and up-to-date summary of therapeutic vaccines for BC. Immunogenic HER2-derived peptide vaccines such as E75 are currently in phase III trial in combination with trastuzumab for the treatment of patients with TNBC, while for GP2, another HER2 glycoprotein-derived peptide vaccine, phase III trial has also been initiated. As mentioned, Globo-H vaccine has shown a trend of superior PFS in phase II and is currently in phase III [101].

Due to chemotherapeutics facing many well know challenges, such as toxicity, poor selectivity, glycoconjugation of drugs has become a promising method for dealing with these issues. Glycoconjugate drugs such as glufosfamide, monosaccharides-conjugated adiamycin, geldanamycin and a significant number of other chemotherapeutics have shown promising effects in cancer therapeutics [102]. Regarding the inhibitors of glycosylation, small molecules interfering with metabolism or intracellular glycosylation processes, such as GM-CT-01 with fluorouracil and fucosylation inhibitor SGN-2FF trials were terminated in phase I for BC, due to various reasons [102].

The aberrant expression of specific GSLs and related enzymes is strongly associated with tumor initiation and malignant transformation malignant transformation. Recently, targeting of glycosphingolipids for cancer immunotherapy has gained increased interest. In 2018, Yu et al. provided a useful summary of the lessons learned in their review on glycosphingolipid targeting for cancer immunotherapy [103]. We have already mentioned the Globo-H vaccine, which is currently being tested. The most recent study examined GD2, a marker for cancer stem cells (CSC), studies suggest, in human TNBC. This study demonstrated that anti-GD2 immunotherapy reduced tumor growth in cell lines using the anti-OAcGD2 antibody mAb8D6, demonstrating the potential of mAb8B6 as a novel immunotherapy for breast CSCs (BCSCs) [104]. A similar recent study examined the function of GD3 synthetase, suggesting a high association of GD3 with EGFR, as well as between the EGFR-inhibitor Gefitinib and the production of GD3. The study suggested GD3 synthetase as a cause of gefitinib resistance, and therefore, represents a potential therapeutic target in breast cancer cells that are EGFR-positive [105].

Finally, besides their potential in the development of novel glycan-based therapy and biomarkers, glycans and compounds involved in glycosylation processes could serve as method of evaluation of efficiency for currently available therapeutic options, such as radiotherapy and help detect radioresistance. After surgery, radiotherapy (RT) is a common adjuvant treatment for BC, since it not only improves local control of cancer development but also lowers the chance of metastatic disease [106]. However, due to radioresistance, a high number of BC patients experience illness recurrence within 10 years of receiving radiation, resulting in poor OS [107,108]. Studies suggest that cancer development has been linked to abnormal expression of various high-mannose type *N*-glycans [6,53]. However, there are also indications that alterations in *N*-glycosylation may be involved in cancer radioresistance. Alpha-1,3-mannosyltransferase (ALG3) is an enzyme located in endoplasmic reticulum and Golgi apparatus and is implicated in post-translational modification of tumor growth factor beta 2 receptor (TGFBR2) by adding mannose as part of the *N*-glycosylation process [109]. A study by Sun X. et al., demonstrated that in patients who had radioresistant BC, their tumor tissues were characterized by an overexpressed *ALG3.* The study showed that radioresistance resulted from an increased glycosylation of TGFBR2. In addition, overexpression of *ALG3* in BC patients was shown to be associated with early local recurrence, poor clinicopathological characteristics and low OS. Study results suggest that ALG3 may be a valuable therapeutic target in BC patients with high ALG3 levels in order to increase radiotherapy effect. ALG3 could also serve as a predictive biomarker of radiotherapy response [10]. In their study, Sun et al., used two compounds to reduce BC radioresistance. The first one was tunicamycin, an antibiotic produced by *Streptomyces lysosuperficus* that is known to inhibit the first step of the *N*-glycosylation and to cause ER stress in certain types of malignant cells leading to their apoptosis and increased sensitivity to chemotherapy [110,111]. Another compound used in this study was LY2109761, a TGFBR2 inhibitor. The study showed that both tunicamycin and LY2109761 lowered cancer radioresistance in BC cells which overexpress ALG3 [10,112]. Additionally, the study showed that tunicamycin can inhibit the migratory and invasive properties of BC cells [112]. Although current data are yet to be extrapolated into clinical context, they are valuable since they represent promising new biomarkers or therapeutic targets that could improve BC patients outcomes.

In the end, after reviewing all these studies and articles showing the potential of glycans and glycosylation processes in diagnostics and therapy, we have to comment on realistic perspectives and successfulness of these approaches. Glycan-based therapy and diagnostics show irrefutable potential with validated results; however, they still require further research, clinical trials and development of clinically efficient assays to provide both diagnostic and therapeutic options to fight BC.

Regarding potential biomarkers, in a recent study by Shipman et al., the authors ask an important question—what are realistic perspectives of all these discoveries and how useful are they from clinical aspect. Although potential glycan biomarkers are being rapidly discovered and their value is supported by a significant amount of evidence, these approaches face difficulties in being adapted into widely implemented diagnostic assays due to their complexity, price, and low throughput. A significant number of studies such as the one mentioned above, enable potential glycan-based biomarkers discoveries to develop into clinically useful assays [113,114]. Another issue with developing clinically practical biomarkers is the need to ensure that they meet specific requirements—sensitivity and specificity. Even the already widely used CA 15-3 biomarker is often not helpful with initial diagnosis, nor in the therapeutic decision-making, although it is highly valuable in evaluating the recurrence of the disease [115]. HER2, a highly valuable and clinically implemented biomarker that is crucial for decision-making regarding therapy, faces the same challenges. Therefore in 2018, American Society of Clinical Oncology/College of American Pathologists published Clinical Practice Guideline Focused Update answering some of the questions regarding sensitivity and specificity, providing guidelines for HER2 testing [116]. Only a few glycan-based cancer biomarkers have been implemented into clinical practice in the last 20 years. This is mainly due to above mentioned reasons, they are less sensitive and specific compared to those already in use or are too complicated to be translated into clinical practice. In their review, Pavlou et al. summarize FDA-approved biomarkers, as well identify a number of issues regarding approving and clinically implementing discovered biomarkers [117].

Regarding the perspective and success of potential glycan-based targeted therapeutic approaches, there is a substantial number of recently FDA-approved glycan-targeting therapies. Monoclonal antibodies targeting HER2 such as trastuzumab (approved in 1998), as well as recently approved pertuzumab and margetuximab, have shown successful results in combination with chemotherapy. Two antibody–drug conjugates (ADCs), meaning a monoclonal antibody conjugated with traditional chemotherapeutic agent, have been approved for BC—trastuzumab emtansine (T-DM1) and trastuzumab deruxtecan (T-DXd), while many other HER2-specific ADCs are undergoing clinical trials. Tyrosine kinase inhibitors, such as lapatinib and tucatinib, that work by inhibiting HER2 downstream signaling, have also recently been approved. For HER2-negative BC patients, including TNBC, monoclonal antibody sacituzumab govitecan (IMMU-132) could be a valuable approved option. It targets trophoblast cell-surface antigen 2 (Trop-2), a type I transmembrane protein post-translationally modified by *N*-linked glycosylation. Vaccines, as already mentioned, are still in various phases of clinical trials [118]. However, therapy targeting glycosylation processes and compounds involved in them is rapidly developing with many recently discovered compounds currently undergoing trials.

## 5. Conclusions

BC treatment efficacy and patient outcomes have improved tremendously in recent years due to discovery of different biomarkers, such as hormone receptors or immune checkpoint receptors and their ligands. However, there is still a significant proportion of patients whose needs remain unmet. For example, patients with TNBC are still in need of more efficient treatment and current knowledge is not sufficient to answer all the uncertainties about treatment failures in some patient groups. The science of glycans is a newer scientific branch which opens a new perspective on intracellular processes involved in malignant transformation and tumor progression. In this review, we summarize the most important knowledge about the glycosylation alterations that are present in BC. Although irrefutably valuable and perspective, specificity and sensitivity, along with complicated process of implementing potential biomarkers in clinical practice remain one of the main challenges and reasons why the rate of discovering biomarkers has not been proportionally followed by their implementation in clinical practice. While also facing difficulties and rigorous clinical trials, the development and approval of glycan-targeted therapy has had significantly greater success, with many recently approved therapeutic options for BC. However, the significance of the review stems from the fact that, in the future, we believe that knowledge about glycosylation changes in BC will aid in the diagnosis, treatment and follow-up of BC. Glycosylation changes may not only be used as biomarkers of response to different treatment modalities, but could also contribute to the discovery of new targeted therapies.

## Figures and Tables

**Table 1:** Glycosylation enzymes, glycans and other glycoconjugates involved in breast cancer progression—potential biomarkers and treatment targets.

	Function	Biomarker/Potential Therapeutic Target	Role in Breast Cancer	Reference
ENZYMES				
Fucosyltransferase 8	Potentiates TGF-β and EGF signaling in BC cells	Biomarker and potential therapeutic target	Promotes BC metastasis and is associated with an aggressive type of BC	[61,62]
2, 6-sialyltransferase	Potentiates BC cancer cell adhesion to ECM	Biomarker and potential therapeutic target	Contributes to BC cell metastatic ability	[25,63]
α2,3-sialyltransferase	Upregulates mediators of malignant invasion: MMP2, COX2 and β1-integrin	Biomarker and potential therapeutic target	Promotes BC metastatic spread	[60]
β-1,3-*N*-acetylglucosaminyl transferase (B3GNT3)	Mediates *N*-glycosylation of PD-L1 glycoprotein	Biomarker and potential therapeutic target	Enables immune evasion in the tumor microenvironment and improves response to immunotherapy	[36,37,38]
Alpha-1,3-mannosyltransferase (ALG3)	*N*-glycosylation of TGFBR2, which promotes radioresistance of BC	Biomarker and potential therapeutic target	Overexpression of ALG3 is associated with BC radioresistance, early local recurrence, poor clinicopathological characteristics and low OS	[7,44]
*N*-acetylgalactosaminyltransferase 6 (GALNT6)	Controls the first stage of mucin-type *O*-glycosylation	Biomarker and potential therapeutic target	BC patients with high GALNT6 expression had worse OS than those with low GALNT6 expression	[12]
TACAs (GLYCANS)				
Thomsen-nouvelle antigen (Tn)	Define various behaviors of cancer cells including apoptosis, alteration of the transmembrane receptor tyrosine kinase pathway, angiogenesis, adhesion, invasion, extravasation and metastases, cell survival.	Biomarker and potential therapeutic target	Overexpressed on BC cells, overexpression associated with increased tumor aggressiveness, lower OS and higher recurrence risks	[66]
sialyl-Thomsen-nouvelle antigen (sTn)				
Thomsen–Friedenreich antigen (TF) antigen				
sialyl-LewisA (sLeA) and sialyl-LewisX (sLeX)	Ligands for adhesion receptors expressed on activated endothelial cells	Biomarker and potential therapeutic target	Promotes penetration into distant tissues	[25,45]
GLYCOPROTEINS				
HER2	Facilitates excessive cell growth and tumorigenesis	Biomarker and potential therapeutic target	Overexpression has a key role in malignant transformation, associated with poor clinical outcomes	[66,67]
PROTEOGLYCANS				
Glypican-3 (GPC3)	Regulates cell proliferation and survival.	Biomarker and potential therapeutic target	Studies suggest a role in inhibition of EMT in BC cells. Requires further elucidation.	[66]

TGF-β—transforming growth factor β; EGF—epidermal growth factor; BC—breast cancer; ECM—extracellular matrix; MMP-2—matrix metalloproteinase-2; COX2—cyclooxygenase-2; PD-L1—programmed death-ligand 1; TGFBR2—transforming growth factor beta receptor II; OS—overall survival; EMT—epithelial-mesenchymal transition.

## Data Availability

Data sharing is not applicable to this article as no new data were created or analyzed in this study.
